# The Impact of COVID-19 on Cancer Screening: Challenges and Opportunities

**DOI:** 10.2196/21697

**Published:** 2020-10-29

**Authors:** Ramon S Cancino, Zhaohui Su, Ruben Mesa, Gail E Tomlinson, Jing Wang

**Affiliations:** 1 Department of Family & Community Medicine Joe R & Teresa Lozano Long School of Medicine UT Health San Antonio San Antonio, TX United States; 2 Center on Smart and Connected Health Technologies Mays Cancer Center School of Nursing, UT Health San Antonio San Antonio, TX United States; 3 Department of Medicine Joe R & Teresa Lozano Long School of Medicine UT Health San Antonio San Antonio, TX United States; 4 Department of Pediatrics Joe R & Teresa Lozano Long School of Medicine UT Health San Antonio San Antonio, TX United States; 5 School of Nursing UT Health San Antonio San Antonio, TX United States

**Keywords:** cancer, screening, COVID-19, coronavirus, telemedicine, social determinants, health, education, training, social media, campaign, branding, cobranding

## Abstract

Cancer is a leading cause of death in the United States and across the globe. Cancer screening is an effective preventive measure that can reduce cancer incidence and mortality. While cancer screening is integral to cancer control and prevention, due to the COVID-19 outbreak many screenings have either been canceled or postponed, leaving a vast number of patients without access to recommended health care services. This disruption to cancer screening services may have a significant impact on patients, health care practitioners, and health systems. In this paper, we aim to offer a comprehensive view of the impact of COVID-19 on cancer screening. We present the challenges COVID-19 has exerted on patients, health care practitioners, and health systems as well as potential opportunities that could help address these challenges.

## Introduction

It is estimated that 606,520 Americans will die from cancer in 2020 [1], which is 4 times the number of recent projected deaths due to COVID-19 [2]. While cancer prevention and screening is integral to personal and population health, the cancer industry is experiencing seismic changes due to the COVID-19 outbreak [3,4]. Disruptions brought by COVID-19 have significantly interrupted almost all aspects of cancer control and prevention infrastructures, including canceled cancer screening services [3], deferred elective surgeries [5], dismantled therapeutic regimens [4], and furloughed health care practitioners [6].

One of the most severely impacted cancer control and prevention services is cancer screening. Cancer screening utilizes medical tests to identify precancerous lesions before cancer is formed or to detect cancer before it progresses into more advanced stages [7,8]. Screening is an effective prevention mechanism that could substantially reduce cancer incidence and mortality rates in patients [9-12]. While not curative, cancer screening has potential to decrease the burden of cancer [13]. Evidence shows that for women of all ages at average risk, screening is linked to an approximate 20% reduction in breast cancer mortality [14]. Data analysis further indicates that 3 times the deaths resulting from colorectal cancer would be avoided with one third of current costs if colorectal cancer screening rates in people aged 50-70 years improved to 80% [15]. For the genetically predisposed individual, the benefit of prescribed cancer screening has an even greater impact [16,17].

Cancer screening plays a critical role in early cancer detection, but COVID-19 has significantly hampered the cancer screening infrastructure [3]. To adjust the provision of health care resources, many cancer agencies have championed the idea of halting cancer screening services to patients [18-20]. After a US national emergency was declared on March 13, 2020, institutions such as the American Cancer Society have made the recommendation that people should pause their cancer screening plans during the COVID-19 outbreak until further notice [19]. This recommendation, along with other contextual factors (eg, social isolation measures), has caused drastic disruptions in cancer screening services. It is estimated that as a result of COVID-19, screenings for cancers of the breast, colon, and cervix have dropped by 94%, 86%, and 94% between January 20, 2020, and April 21, 2020, respectively [21]. Little is known about the impact the current pandemic will have on the cancer screening and prevention activities of patients, health care practitioners, and health systems. To bridge this gap, we aim to present the challenges COVID-19 has exerted on patients, health care practitioners, and health systems as well as potential opportunities that could help address these challenges.

## Cancer Screening Challenges, Opportunities, and Solutions

**Figure 1:**
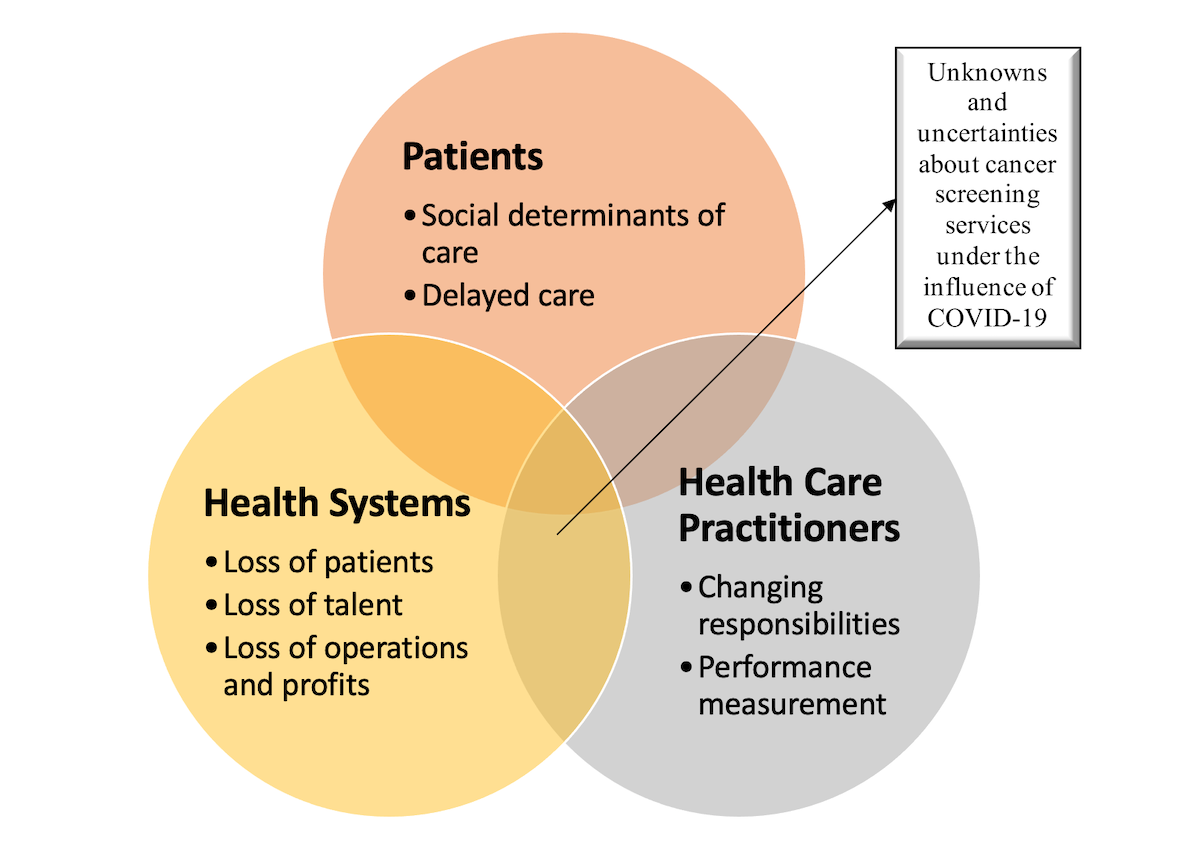
Summary of challenges patients, health care professionals, and health systems face due to COVID-19.

Successful cancer screening is often carried out as a result of synergistic collaborations between patients, health care practitioners, and health systems [22-24]. Furthermore, as no evidence is available on the origin of the virus and no effective vaccine or curative medicine is available, both patients and health care practitioners also experience the shared unknowns and uncertainties regarding COVID-19. These uncertainties are also experienced by health systems, whose financial futures may be threatened. Therefore, to acknowledge the shared interests of patients, health care practitioners, and health systems in cancer screening, we organized evidence and insights around these key stakeholders to provide a connected and comprehensive understanding of the impact of COVID-19 on cancer screening (Figure 1).

### Patients

Social determinants of health could be understood as the condition in which people are born, grow, live, work, and age [25]. In other words, as opposed to biological factors (eg, genetic traits), social determinants of health are a range of social, economic, political, and environmental factors that contribute to individuals’ health conditions and disparities, such as inequalities in cancer screening [26-28]. Results show that patients who have poor social determinants of health, such as lack of insurance, low income, and living in a deprived neighborhood, are often less likely to adopt cancer screening [28-30]. Evidence from randomized clinical trials further indicates that, compared to patients with private insurance, patients with Medicaid or with no insurance received reduced benefits from the same intervention program [31]. These combined insights may help explain why screenings for cancers have dropped significantly since January 2020 (eg, breast cancer screening has dropped by 94%) [21]. The experience of dramatic events, such as the COVID-19 pandemic, losing health insurance, and lack of access to health care, and in some situations caring for ill family members, may exert added psychological pressure on patients and further impact the ability to receive services and increase their risk of medical conditions such as cancer [32,33].

Another social determinant of health, economic stability, has been greatly affected. Due to the impact of COVID-19, unemployment rates rose to a historical 20.6% in the United States, with more than 31 million workers filing unemployment claims between March 1, 2020, and May 2, 2020 [34]. It is estimated that 26.6 million workers and their dependents may lose their employer-based insurance [35]. This undoubtedly can have a detrimental effect on individuals’ physical and psychological health, as health insurance status is often considered as a key social determinant of health that has substantial influence on individuals’ ability to access health care services [36,37].

Canceling or postponing cancer screenings may not equate to avoiding a cancer diagnosis but delayed cancer diagnoses could lead to increased mortality. On the contrary, the drastic decrease of cancer screenings in the United States and across the globe may have severe consequences, such as an unexpected rise in cancer incidence and later-stage cancer diagnosis, and in turn, more cancer deaths in patients [11,38-41]. While patients might be in great need for help during this crisis, assistance from health care practitioners was also interrupted due to the COVID-19 pandemic [42]. Furthermore, the accumulated need to screen those patients whose exams or procedures were postponed could directly impact *other* patients whose exams or procedures are now also due, creating downstream cancer screening delays.

### Health Care Practitioners

One of the most impacted populations by COVID-19 is the health care practitioner community [43,44]. During the SARS (severe acute respiratory syndrome) outbreak, health care workers and hospital systems experienced measurable negative psychological impacts [45-48]. Due to the current outbreak, health care practitioners may have experienced a variety of multilevel stressors, such as (1) interruptions in routine job duties and responsibilities, (2) limited knowledge and data, and (3) worries about job security due to decreased patient volumes. COVID-19 has caused significant upheavals in the cancer health care infrastructure, including disturbed clinical visits, canceled or delayed medical surgery or procedures, and bridled therapeutic strategies [44,49]. For health care practitioners, these changes force them to tackle constant unexpected disruptions to routine job duties and responsibilities, such as the need to quickly learn and adopt telemedicine tools until COVID-19 ceases to be a threat to society. This unexpected need to adopt telemedicine may cause stress in health care practitioners, as some of them may be forced into adopting technology-based health solutions without necessary knowledge or adequate training in place [50]. These changes in job duties and responsibilities may put extra pressure on health care practitioners, above and beyond the levels of stress experienced by the general public in the face of COVID-19. For some health care practitioners, in addition to the unique work requirements and responsibilities they shoulder during the COVID-19 pandemic, the fear of being exposed to SARS-CoV-2 at work may cause additional stress and anxiety [43]. This, in turn, may cause detrimental consequences on their psychological health and their performance in administering cancer care and treatment to patients.

Without key information from insurance payers, health care practitioners may lack the necessary data needed to identify those who need cancer screening [27,51]. Though many health care practitioners have access to electronic health record systems, information stored in these systems is often too outdated and inaccurate to be utilized [51,52]. This suggests that limited data may also hamper health practitioners’ ability to help patients. Therefore, due to these issues coupled with COVID-19–related cancer screening cancelations and delays [21], health care professionals’ performance in value-based contracts are at risk [53]. One consequence could be decreased screening rates and the resulting poor performance in cancer screening metrics, which in turn can lead to decreased quality incentives [54,55].

Reduced successful cancer care could be manifested in terms of decreased profits and diminished research funding [53], which may then result in downstream cost reduction and job loss. As a matter of fact, health care institutions, including hospitals and nonprofit organizations, such as the American Cancer Society, have been downsizing in the form of furloughs and layoffs [6]. According to the Labor Department, 1.4 million health care practitioners have lost their job since January 2020 [56]. This grim job reality could exert additional pressure to the unknowns and uncertainties health care practitioners are facing while trying to protect themselves and patients from COVID-19.

### Health Systems

In the context of cancer screening, the impact of COVID-19 on health systems can be best illustrated in terms of loss: (1) loss of lives, (2) loss of talent, and (3) loss of operational activity and revenues. Globally, it is estimated that 2,324,069 elective cancer surgeries (37.7% of all 1,735,483 elective surgical operations) were canceled or postponed during the 12-week peak disruptions caused by COVID-19 [5]. These cancelations and delays could cause cancer disparities to become more pronounced. It is difficult to know how these discontinued services could further negatively impact the patient-provider relationship.

It is also hard to predict how patients will respond to cancer screening messages from health care practitioners post COVID-19. Public perception of health care safety could impact utilization patterns of health care [57-59]. Since COVID-19 is seen as highly infectious and can be contracted from direct contact with others [60,61], it is possible that the current avoidance of health care may continue and patients without symptoms may opt to not be screened for preventive care. This could have a detrimental effect on patients’ health, as many chronic medical condition such as cancer, high blood pressure, and diabetes are often asymptomatic until needing urgent attention [62-64]. Furthermore, drastic changes in patients’ social determinants of health (eg, health insurance status, geographic distance from health care center and associated transportation needs, etc) may also contribute to the development of other non–cancer-related illnesses [65-68], resulting in competing interests in health care decisions that could further dampen patients’ motivation to seek cancer screening services [69]. This, in turn, may also contribute to an increase of later-stage cancer diagnosis in patients. Early data from the United Kingdom predicts a substantial increase in the number of avoidable cancer-related deaths in England [70]. Other estimates predict COVID-19 will result in 10,000 excess deaths from breast and colorectal cancer [71].

Health care practitioners are losing their jobs, partially due to the dwindled demands for health care services caused by COVID-19. Overall, 1.4 million health care practitioners lost their jobs since January 2020 [56]. Though the potential impact of COVID-19 on medical and nursing school enrollments is yet to be ascertained, it is possible that COVID-19 may have a negative impact on health care practitioners’ ability to provide high-quality education. Moreover, the impact of reduced patient contact and virtual learning on educational milestone attainment are yet to be determined.

While it is difficult to pinpoint the exact impact of disrupted cancer screening services on the loss of life or loss of talent in the health care industry, it is easier to describe the decreased activity and estimated the loss of profits in the health care industry caused by COVID-19. According to the American Hospital Association, due to the impact of this coronavirus, the estimated loss of US hospitals and health systems between February 2020 and June 2020 would amount to $202.6 billion [53]. This loss of profit may also have an impact on patients and health care practitioners, considering that loss of profits often translate into reduced investments in cancer research [6]. However, while these numbers present a dismal reality, opportunities and solutions that could address the challenges caused by COVID-19 on cancer screening are also available.

The impact of these health system issues on cancer screening measures are coming to light, especially as it relates to cancer screening. The World Health Organization warned of a worldwide decrease in health services for noncommunicable diseases [72]. These results include predicted increases in avoidable cancer deaths [70]. Many health care systems are finding fewer cancer diagnoses during the pandemic [73,74]. Fewer diagnoses can have financial impact on health care systems, especially when the United States spent roughly $87.8 billion on cancer-related health care in 2014 [75].

## Cancer Screening for At-Risk Patient Populations

We also need to pay attention to where the COVID-19 pandemic hit hardest and where cancer screening rates are the lowest in our community [76-85]. Patients with low socioeconomic status (SES) or identify as minority, including racial and ethnic underserved minorities such as Hispanics and African Americans, and the LGBTQ (lesbian, gay, bisexual, transgender, and queer or questioning) sexual and gender minorities. It is important to recognize that there is a huge overlap between patients with low SES and those with minority status—rather than face the double impact of being poor and disenfranchised (eg, heightened risks for cancer) [84,86-88], as a result of COVID-19, they now face the additional impact of the need to pay extra attention and allocate already limited resources to protect themselves against the coronavirus while also tackling unemployment or hazardous working conditions [89-91].

It is important to note that the impact of missing a cancer screening is not the same for every population [92,93]. Evidence suggests that marginalized individuals such as racial minorities are more likely to benefit from cancer screening [94]. Research also indicates that cancer screening is more cost-effective for high-risk races and ethnicities, such as Asians ($71,451 per quality-adjusted life year [QALY]), Hispanics ($76,070/QALY), African Americans ($80,278/QALY), compared to non-Hispanic White individuals ($122,428/QALY) [95]. While these findings further support the importance of cancer screening, they also indicate that the likelihood of missing a diagnosis by delayed or missed screening will be amplified among these minority populations. In other words, screening is integral to these populations’ protection against cancer.

COVID-19 has also helped expose many health disparities minorities face, especially structured and systematic health inequalities such as violence against women [77,96-99]. Prior to COVID-19, data from the World Health Organization already painted a horrifying picture where 1 in 3 women will become a victim of sexual or physical violence in a relationship at some point in their life [100]. A growing body of literature suggests that, as the pandemic and lockdown measures bring continuing financial blows and forced “close” time with their partners, women worldwide are experiencing more frequent and dangerous forms of abuse [77,96,98].

With so many people taking a stand and making their voice heard over injustice, as exemplified by the belated realization of police brutality in the United States, there is a societal need to pay attention to the disparities and inequalities that, we, as a population, are experiencing on a daily basis. “Pay inequity” [101] or “violence against women” [77,100] are more than inhumane terminologies or irrelevant phenomena to leave as inheritance for future generations—rather, these disparities are negatively impacting our grandmothers, mothers, and daughters’ well-being and making them less likely to screen for cancer [102,103] and thus more at risk for missing early cancer detection [104-106]. It is questionable as to how likely a woman experiencing domestic violence will undertake the initiative to screen for breast cancer amid the pandemic, even if she is aware that lumps in her breasts have appeared or changed. The ramifications of COVID-19 are thus profound.

More attention from health care practitioners are required to address these issues while improving screening rates for the highest at-risk populations. In other words, these health disparities that minorities face are meaningful and life-or-death facts that health care practitioners must acknowledge and address.

Some of the approaches to more universal access to cancer screening using traditional and organized outreach measures include local mammography vans for breast cancer [38,107], fecal immunochemical test (FIT) or other at-home stool tests for colon cancer detection [108,109], and cost-effective technology-based solutions such as social media campaigns [110,111], so that a broader population can be served and the widening cancer disparities can be alleviated. In the fight against inequalities, preventative measures such as cancer screening are more relevant to underserved populations than ever before. Since COVID-19 is more likely to be deadly for marginalized individuals with chronic conditions and cancer [112-116], it is important to ensure people can fight to overcome social determinants and injustices with maintaining a healthy and cancer-free body.

## Opportunities and Solutions

### Telemedicine Opportunities and Technology-Based Solutions

With the advances in science and technology, the application of telemedicine in cancer care and management is gaining momentum [117-119]. Telemedicine, which literally means “healing at a distance” [120], could be understood as the delivery of health care services aiming to advance personal and population health [121]. Telemedicine allows timely, accessible, and cost-effective health care delivery to the patients, which renders itself a practical solution to COVID-19–induced constraints such as social distancing and self-isolation [122-124]. Telemedicine tools such as virtual reality devices have been found to be useful for training health care practitioners [125]. As virtual reality can offer remote yet realistic training experiences, it facilitates training for health care professionals in a time when social isolation is the norm. Telemedicine has been shown to be effective in underserved geographically remote populations. Emerging technologies such as artificial intelligence (AI) also have great potential in facilitating cancer screening [119].

On a higher-technological scale, using a deep learning technique, researchers found that AI can help identify faces of patients with cancer from those without [126]. This promising finding, not currently in use, suggests that AI-based telemedicine tools have the future potential to assist patients and health care practitioners with cancer screening and improve screening accuracy.

While promising telemedicine opportunities are present, to successfully implement telemedicine in cancer care and primary care, education and training should be made available to both patients and health care practitioners [118]. Research conducted by Stanford University shows that 47% of physicians and 73% of medical students surveyed indicated that they are considering taking additional courses to better prepare for innovations in health care (eg, data science, AI) [127]. While it is imperative to update college curricula to reflect health care needs identified in practice [128,129], it is important to note that telemedicine education and training should be considered as a long-term investment, rather than a short-term experiment. In other words, as technology advances, telemedicine education and training programs should also be updated regularly and frequently to ensure health care practitioners are up to date with telemedicine opportunities for the benefits of self and patients [130,131].

According to the Pew Research Center, approximately 96% of Americans own a cellphone of some kind [132]. Considering the prevalence of smart devices patients own, health care practitioners may face questions like “Which mobile apps can help me better take care of my health?” from patients more frequently in the future. There is also a boom in the medical app market. It was estimated that there were approximately 325,000 health apps available to patients in 2017, equating to 3.7 billion app downloads in total [133]. As mobile health (mHealth) continues to gather momentum, health care practitioners may also need to “prescribe” mobile apps to patients to protect them from ill-suited (eg, apps addressing different sets of needs) or poorly developed apps (eg, apps filled with misinformation or lack of scientific underpinning) [134]. Technology competence might be an integral part to effective patient-provider communication [123]. To embrace future technology-based health care challenges, health care practitioners may have to train their telemedicine muscles with regular education to be able to adequately answer patients’ questions and concerns about telemedicine.

### Leveraging Social Media to Boost Cancer Screening

In addition to boosting health care professionals’ core competence with regard to telemedicine [131], health systems should also consider adopting integrated marketing campaigns, such as social media campaigns, to increase screening awareness and adoption rates in patients. Social media campaigns could be understood as the use of social media platforms to deliver persuasive communication strategies to the target audience in order to change their attitudes and behavior to improve health. One key advantage of social media campaigns is that as persuasive strategies adopted in these campaigns are evidence-based and tailored to the target audience [135,136], they often yield desirable campaign outcomes [137-139].

Social media campaigns may be extremely useful for promoting cancer screening services to at-risk populations. Compared to integrated marketing campaigns distributed via traditional media platforms, social media campaigns can be distributed remotely with limited costs and therefore have the added advantages of cost-effectiveness and scalability [135,136]. This advantage might be more pronounced in the era of COVID-19; since lockdowns and social distancing measures have limited people’s ability to physically disseminate campaign messages, campaign mechanisms that can virtually distribute promotional information are desired. Evidence suggests that social media campaigns are effective in raising cancer screening awareness in the target audience [110,111,140]. Promising findings show that social media campaigns on lung cancer screening using Google and Facebook to reach at-risk populations yielded click-through rates above the industry standard [110]. These insights suggest that health care professionals can consider using social media campaigns to reach at-risk populations, such as minorities with pronounced needs to be screened for cancer, to further address the widening cancer disparities exacerbated by COVID-19.

## Conclusion

The systemic disruption and tragedy that COVID-19 has brought to patients, practitioners, and health care systems is an opportunity for innovative solutions, especially in cancer prevention and screening [141-143]. Cancer prevention and screening professionals need to innovate in this current environment to continue to decrease the burden of cancer in communities. We need agile short-term plans tailored to the current COVID-19 infection control strategies as well as long-term plans that account for the capricious, costly, and deadly nature of cancer and its intersection with other widespread health problems, such as viral infections similar to the current pandemic. We offer some post–COVID-19 screening enhancement recommendations below:

- Breast cancer screening
  Mobile mammography unit
- Cervical cancer screening
  Pap smears +/– cotesting per guidelines
- Colon cancer screening
  Enhanced workflows for FIT or Cologuard with appropriate patients
- General solutions:
  Proactive outreach to patients due for screening
  Social media communication to patients about risks of cancer and safety of screening procedures
  Initial assessment and results follow-up via telemedicine appointment
  Masking precautions (patient, clinician, and staff)
  Social distancing precautions when possible

Complacency is not an option, and health care professionals must diligently work together with other stakeholders and across disciplines toward solutions to ensure patients, providers, and health systems have the tools and means necessary to screen for cancer *now*.

## Abbreviations

AI: artificial intelligence
FIT: fecal immunochemical test
LGBTQ: lesbian, gay, bisexual, transgender, and queer or questioning
mHealth: mobile health
QALY: quality-adjusted life year
SARS: severe acute respiratory syndrome
SES: socioeconomic status

## References

[1] Siegel R, Miller K, Jemal A (2020). Cancer statistics, 2020. CA Cancer J Clin.

[2] (2020). COVID-19 forecasts. Centers for Disease Control and Prevention.

[3] Waterhouse DM, Harvey RD, Hurley P, Levit LA, Kim ES, Klepin HD, Mileham KF, Nowakowski G, Schenkel C, Davis C, Bruinooge SS, Schilsky RL (2020). Early Impact of COVID-19 on the Conduct of Oncology Clinical Trials and Long-Term Opportunities for Transformation: Findings From an American Society of Clinical Oncology Survey. JCO Oncology Practice.

[4] Ren X, Chen B, Hong Y, Liu W, Jiang Q, Yang J, Qian Q, Jiang C (2020). The challenges in colorectal cancer management during COVID-19 epidemic. Ann Transl Med.

[5] CovidSurg Collaborative (2020). Elective surgery cancellations due to the COVID-19 pandemic: global predictive modelling to inform surgical recovery plans. Br J Surg.

[6] American Association for Cancer Research (2020). COVID-19 Hits Cancer Research Funding. Cancer Discov.

[7] Cancer: Screening. World Health Organization.

[8] Division of Cancer Prevention and Control (2020). Screening tests. Centers for Disease Control and Prevention.

[9] Lieberman D, Sullivan BA, Hauser ER, Qin X, Musselwhite LW, O'Leary Meghan C, Redding TS, Madison AN, Bullard AJ, Thomas R, Sims KJ, Williams CD, Hyslop T, Weiss D, Gupta S, Gellad ZF, Robertson DJ, Provenzale D (2020). Baseline Colonoscopy Findings Associated With 10-Year Outcomes in a Screening Cohort Undergoing Colonoscopy Surveillance. Gastroenterology.

[10] Ran T, Cheng C, Misselwitz B, Brenner H, Ubels J, Schlander M (2019). Cost-Effectiveness of Colorectal Cancer Screening Strategies-A Systematic Review. Clin Gastroenterol Hepatol.

[11] Miller EA, Pinsky PF, Schoen RE, Prorok PC, Church TR (2019). Effect of flexible sigmoidoscopy screening on colorectal cancer incidence and mortality: long-term follow-up of the randomised US PLCO cancer screening trial.

[12] Hallowell BD, Endeshaw M, McKenna MT, Senkomago V, Razzaghi H, Saraiya M (2019). Cervical Cancer Death Rates Among U.S.- and Foreign-Born Women: U.S., 2005-2014. Am J Prev Med.

[13] Curry Susan J, Byers T, Hewitt M, Institute of Medicine (US) and National Research Council (US) National Cancer Policy Board (2003). Fulfilling the Potential of Cancer Prevention and Early Detection.

[14] Myers ER, Moorman P, Gierisch JM, Havrilesky LJ, Grimm LJ, Ghate S, Davidson B, Mongtomery RC, Crowley MJ, McCrory DC, Kendrick A, Sanders GD (2015). Benefits and Harms of Breast Cancer Screening: A Systematic Review. JAMA.

[15] Ladabaum U, Mannalithara A, Meester RG, Gupta S, Schoen RE (2019). Cost-Effectiveness and National Effects of Initiating Colorectal Cancer Screening for Average-Risk Persons at Age 45 Years Instead of 50 Years. Gastroenterology.

[16] Gupta S, Provenzale D, Regenbogen SE, Hampel H, Slavin TP, Hall MJ, Llor X, Chung DC, Ahnen DJ, Bray T, Cooper G, Early DS, Ford JM, Giardiello FM, Grady W, Halverson AL, Hamilton SR, Klapman JB, Larson DW, Lazenby AJ, Lynch PM, Markowitz AJ, Mayer RJ, Ness RM, Samadder NJ, Shike M, Sugandha S, Weiss JM, Dwyer MA, Ogba N (2017). NCCN Guidelines Insights: Genetic/Familial High-Risk Assessment: Colorectal, Version 3.2017. J Natl Compr Canc Netw.

[17] Daly M, Pilarski R, Yurgelun M, Berry Michael P, Buys Saundra S, Dickson Patricia, Domchek Susan M, Elkhanany Ahmed, Friedman Susan, Garber Judy E, Goggins Michael, Hutton Mollie L, Khan Seema, Klein Catherine, Kohlmann Wendy, Kurian Allison W, Laronga Christine, Litton Jennifer K, Mak Julie S, Menendez Carolyn S, Merajver Sofia D, Norquist Barbara S, Offit Kenneth, Pal Tuya, Pederson Holly J, Reiser Gwen, Shannon Kristen Mahoney, Visvanathan Kala, Weitzel Jeffrey N, Wick Myra J, Wisinski Kari B, Dwyer Mary A, Darlow Susan D (2020). NCCN Guidelines Insights: Genetic/Familial High-Risk Assessment: Breast, Ovarian, and Pancreatic, Version 1.2020. J Natl Compr Canc Netw.

[18] (2020). ASBrS and ACR joint statement on breast screening exams during the COVID-19 pandemic. The American Society of Breast Surgeons.

[19] Falco M (2020). Common questions about the new coronavirus outbreak. American Cancer Society.

[20] (2020). ASCCP interim guidance for timing of diagnostic and treatment procedures for patients with abnormal cervical screening test. American Society for Colposcopy and Cervical Pathology.

[21] (2020). Preventive cancer screenings during COVID-19 pandemic. Epic Health Research Network.

[22] Warner ET, Lathan CS (2019). Race and sex differences in patient provider communication and awareness of lung cancer screening in the health information National Trends Survey, 2013-2017. Prev Med.

[23] Williamson S, Patterson J, Crosby R, Johnson R, Sandhu H, Johnson S, Jenkins J, Casey M, Kearins O, Taylor-Phillips S (2019). Communication of cancer screening results by letter, telephone or in person: A mixed methods systematic review of the effect on attendee anxiety, understanding and preferences. Prev Med Rep.

[24] Sava MG, Dolan JG, May JH, Vargas LG (2018). A personalized approach of patient-health care provider communication regarding colorectal cancer screening options. Med Decis Making.

[25] Social determinants of health. World Health Organization.

[26] Mosquera I, Mendizabal N, Martín Unai, Bacigalupe Amaia, Aldasoro Elena, Portillo Isabel, from the Desberdinak Group (2020). Inequalities in participation in colorectal cancer screening programmes: a systematic review. Eur J Public Health.

[27] Abdelsattar ZM, Hendren S, Wong SL (2017). The impact of health insurance on cancer care in disadvantaged communities. Cancer.

[28] Lofters A, Schuler A, Slater M, Baxter N, Persaud N, Pinto A, Kucharski E, Davie S, Nisenbaum R, Kiran T (2017). Using self-reported data on the social determinants of health in primary care to identify cancer screening disparities: opportunities and challenges. BMC Fam Pract.

[29] Kurani SS, McCoy RG, Lampman MA, Doubeni CA, Finney Rutten LJ, Inselman JW, Giblon RE, Bunkers KS, Stroebel RJ, Rushlow D, Chawla SS, Shah ND (2020). Association of Neighborhood Measures of Social Determinants of Health With Breast, Cervical, and Colorectal Cancer Screening Rates in the US Midwest. JAMA Netw Open.

[30] Coughlin SS (2020). Social determinants of colorectal cancer risk, stage, and survival: a systematic review. Int J Colorectal Dis.

[31] Unger JM, Blanke CD, LeBlanc M, Barlow WE, Vaidya R, Ramsey SD, Hershman DL (2020). Association of Patient Demographic Characteristics and Insurance Status With Survival in Cancer Randomized Clinical Trials With Positive Findings. JAMA Netw Open.

[32] Man RX, Lack DA, Wyatt CE, Murray V (2018). The effect of natural disasters on cancer care: a systematic review. The Lancet Oncology.

[33] Prohaska T, Peters K (2019). Impact of Natural Disasters on Health Outcomes and Cancer Among Older Adults. Gerontologist.

[34] (2020). Unemployment insurance weekly claims. US Department of Labor.

[35] Garfield R, Claxton G, Damico A, Levitt L (2020). Eligibility for ACA health coverage following job loss. Kaiser Family Foundation.

[36] Smith MA, Weiss JM, Potvien A, Schumacher JR, Gangnon RE, Kim DH, Weeth-Feinstein LA, Pickhardt PJ (2017). Insurance Coverage for CT Colonography Screening: Impact on Overall Colorectal Cancer Screening Rates. Radiology.

[37] Moss JL, Murphy J, Filiaci VL, Wenzel LB, Minasian L, Temkin SM (2019). Disparities in health-related quality of life in women undergoing treatment for advanced ovarian cancer: the role of individual-level and contextual social determinants. Support Care Cancer.

[38] Vang S, Margolies LR, Jandorf L (2018). Mobile Mammography Participation Among Medically Underserved Women: A Systematic Review. Prev Chronic Dis.

[39] Tarver WL, Haggstrom DA (2019). The Use of Cancer-Specific Patient-Centered Technologies Among Underserved Populations in the United States: Systematic Review. J Med Internet Res.

[40] Houghton LC, Howland RE, McDonald JA (2019). Mobilizing Breast Cancer Prevention Research Through Smartphone Apps: A Systematic Review of the Literature. Front Public Health.

[41] Uy C, Lopez J, Trinh-Shevrin C, Kwon S, Sherman S, Liang P (2017). Text Messaging Interventions on Cancer Screening Rates: A Systematic Review. J Med Internet Res.

[42] Romeo A, Castelli L, Franco P (2020). The Effect of COVID-19 on Radiation Oncology Professionals and Patients With Cancer: From Trauma to Psychological Growth. Adv Radiat Oncol.

[43] Shanafelt T, Ripp J, Trockel M (2020). Understanding and Addressing Sources of Anxiety Among Health Care Professionals During the COVID-19 Pandemic. JAMA.

[44] Desideri I, Pilleron S, Battisti NML, Gomes F, de Glas N, Neuendorff NR, Liposits G, Paredero-Pérez Irene, Lok WCW, Loh KP, DuMontier C, Mian H, Soto-Perez-de-Celis E (2020). Caring for older patients with cancer during the COVID-19 pandemic: A Young International Society of Geriatric Oncology (SIOG) global perspective. J Geriatr Oncol.

[45] Maunder R, Lancee W, Balderson K, Bennett J, Borgundvaag B, Evans S, Fernandes C, Goldbloom D, Gupta M, Hunter J, McGillis Hall L, Nagle L, Pain C, Peczeniuk S, Raymond G, Read N, Rourke S, Steinberg R, Stewart T, VanDeVelde-Coke S, Veldhorst G, Wasylenki D (2006). Long-term psychological and occupational effects of providing hospital healthcare during SARS outbreak. Emerg Infect Dis.

[46] Maunder R, Hunter J, Vincent L, Bennett J, Peladeau N, Leszcz M, Sadavoy J, Verhaeghe LM, Steinberg R, Mazzulli T (2003). The immediate psychological and occupational impact of the 2003 SARS outbreak in a teaching hospital. CMAJ.

[47] Grace SL, Hershenfield K, Robertson E, Stewart DE (2005). The occupational and psychosocial impact of SARS on academic physicians in three affected hospitals. Psychosomatics.

[48] Chu D, Chen R, Ku C, Chou P (2008). The impact of SARS on hospital performance. BMC Health Serv Res.

[49] Segelov E, Underhill C, Prenen H, Karapetis C, Jackson C, Nott L, Clay T, Pavlakis N, Sabesan S, Heywood E, Steer C, Lethborg C, Gan HK, Yip D, Karanth N, Karikios D, MacIntyre CR (2020). Practical Considerations for Treating Patients With Cancer in the COVID-19 Pandemic. JCO Oncology Practice.

[50] Meti N, Rossos PG, Cheung MC, Singh S (2020). Virtual Cancer Care During and Beyond the COVID-19 Pandemic: We Need to Get It Right. JCO Oncology Practice.

[51] Cole AM, Pflugeisen B, Schwartz MR, Miller SC (2018). Cross sectional study to assess the accuracy of electronic health record data to identify patients in need of lung cancer screening. BMC Res Notes.

[52] Marino M, Angier H, Valenzuela S, Hoopes M, Killerby M, Blackburn B, Huguet N, Heintzman J, Hatch B, O'Malley JP, DeVoe JE (2018). Medicaid coverage accuracy in electronic health records. Prev Med Rep.

[53] (2020). Hospitals and health systems face unprecedented financial pressures due to COVID-19. American Hospital Association.

[54] Petersen LA, Woodard LD, Urech T, Daw C, Sookanan S (2006). Does pay-for-performance improve the quality of health care?. Ann Intern Med.

[55] Green E, Peterson KS, Markiewicz K, O'Brien J, Arring NM (2020). Cautionary study on the effects of pay for performance on quality of care: a pilot randomised controlled trial using standardised patients. BMJ Qual Saf.

[56] (2020). The employment situation—September 2020. US Bureau of Labor Statistics.

[57] Bednarczyk RA, Chu SL, Sickler H, Shaw J, Nadeau JA, McNutt L (2015). Low uptake of influenza vaccine among university students: Evaluating predictors beyond cost and safety concerns. Vaccine.

[58] Cronholm PF, Bowman MA (2009). Women with safety concerns report fewer gender-specific preventive healthcare services. J Womens Health (Larchmt).

[59] Akbar S, Coiera E, Magrabi F (2020). Safety concerns with consumer-facing mobile health applications and their consequences: a scoping review. J Am Med Inform Assoc.

[60] Sun P, Lu X, Xu C, Sun W, Pan B (2020). Understanding of COVID-19 based on current evidence. J Med Virol.

[61] Dowd JB, Andriano L, Brazel DM, Rotondi V, Block P, Ding X, Liu Y, Mills MC (2020). Demographic science aids in understanding the spread and fatality rates of COVID-19. Proc Natl Acad Sci U S A.

[62] Muggah E, Graves E, Bennett C, Manuel DG (2013). Ascertainment of chronic diseases using population health data: a comparison of health administrative data and patient self-report. BMC Public Health.

[63] Beaglehole R, Epping-Jordan J, Patel V, Chopra M, Ebrahim S, Kidd M, Haines A (2008). Improving the prevention and management of chronic disease in low-income and middle-income countries: a priority for primary health care. The Lancet.

[64] Wendimagegn NF, Bezuidenhout M (2019). The integrated health service model: the approach to restrain the vicious cycle to chronic diseases. BMC Health Serv Res.

[65] Nomura S, Parsons AJ, Hirabayashi M, Kinoshita R, Liao Y, Hodgson S (2016). Social determinants of mid- to long-term disaster impacts on health: A systematic review. International Journal of Disaster Risk Reduction.

[66] Prüss-Ustün A, Wolf J, Corvalán C, Neville T, Bos R, Neira M (2017). Diseases due to unhealthy environments: an updated estimate of the global burden of disease attributable to environmental determinants of health. J Public Health (Oxf).

[67] Ruckert A, Labonté Ronald (2017). Health inequities in the age of austerity: The need for social protection policies. Soc Sci Med.

[68] Cockerham WC, Hamby BW, Oates GR (2017). The Social Determinants of Chronic Disease. Am J Prev Med.

[69] Rosland A, Kieffer EC, Tipirneni R, Kullgren JT, Kirch M, Arntson EK, Clark SJ, Lee S, Solway E, Beathard E, Ayanian JZ, Goold SD (2019). Diagnosis and Care of Chronic Health Conditions Among Medicaid Expansion Enrollees: a Mixed-Methods Observational Study. J Gen Intern Med.

[70] Maringe C, Spicer J, Morris M, Purushotham A, Nolte E, Sullivan R, Rachet B, Aggarwal A (2020). The impact of the COVID-19 pandemic on cancer deaths due to delays in diagnosis in England, UK: a national, population-based, modelling study. The Lancet Oncology.

[71] Sharpless NE (2020). COVID-19 and cancer. Science.

[72] (2020). COVID-19 significantly impacts health services for noncommunicable diseases. World Health Organization.

[73] Kaufman HW, Chen Z, Niles J, Fesko Y (2020). Changes in the Number of US Patients With Newly Identified Cancer Before and During the Coronavirus Disease 2019 (COVID-19) Pandemic. JAMA Netw Open.

[74] Dinmohamed AG, Visser O, Verhoeven RHA, Louwman MWJ, van Nederveen FH, Willems SM, Merkx MAW, Lemmens VEPP, Nagtegaal ID, Siesling S (2020). Fewer cancer diagnoses during the COVID-19 epidemic in the Netherlands. The Lancet Oncology.

[75] Total Expenses and Percent Distribution Selected Conditions by Source of Payment: United States, 2014 (generated interactively). Agency for Healthcare Research and Quality.

[76] Chowkwanyun M, Reed AL (2020). Racial Health Disparities and Covid-19 — Caution and Context. N Engl J Med.

[77] United Nations Development Programme (2020). Gender-based violence and COVID-19. United Nations.

[78] Bhala N, Curry G, Martineau AR, Agyemang C, Bhopal R (2020). Sharpening the global focus on ethnicity and race in the time of COVID-19. The Lancet.

[79] Aldridge RW, Lewer D, Katikireddi SV, Mathur R, Pathak N, Burns R, Fragaszy EB, Johnson AM, Devakumar D, Abubakar I, Hayward A (2020). Black, Asian and Minority Ethnic groups in England are at increased risk of death from COVID-19: indirect standardisation of NHS mortality data. Wellcome Open Res.

[80] Nardi C, Sandhu P, Selix N (2016). Cervical Cancer Screening Among Minorities in the United States. The Journal for Nurse Practitioners.

[81] Carter-Harris L, Slaven JE, Monahan PO, Shedd-Steele R, Hanna N, Rawl SM (2018). Understanding lung cancer screening behavior: Racial, gender, and geographic differences among Indiana long-term smokers. Prev Med Rep.

[82] Tabaac AR, Sutter ME, Wall CS, Baker KE (2018). Gender Identity Disparities in Cancer Screening Behaviors. Am J Prev Med.

[83] Eke R, Tariq T, Li T, Irfan FB (2019). Colorectal cancer screening in hospitalized patients: results from the Nationwide Inpatient Sample. European Journal of Cancer Prevention.

[84] Akinlotan M, Bolin JN, Helduser J, Ojinnaka C, Lichorad A, McClellan D (2017). Cervical Cancer Screening Barriers and Risk Factor Knowledge Among Uninsured Women. J Community Health.

[85] Sharma KP, DeGroff A, Scott L, Shrestha S, Melillo S, Sabatino SA (2019). Correlates of colorectal cancer screening rates in primary care clinics serving low income, medically underserved populations. Prev Med.

[86] Efuni E, Schofield E, Duhamel KN, Villagra C, Cohen N, Reid F, Jandorf L (2018). Optimism, Worry, and Colorectal Cancer Screening among Low-income Latinos. Health Behavior and Policy Review.

[87] Liang W, Guan W, Chen R, Wang W, Li J, Xu K, Li C, Ai Q, Lu W, Liang H, Li S, He J (2020). Cancer patients in SARS-CoV-2 infection: a nationwide analysis in China. The Lancet Oncology.

[88] Mandelblatt J, Andrews H, Kao R, Wallace R, Kerner J (1996). The late-stage diagnosis of colorectal cancer: demographic and socioeconomic factors. Am J Public Health.

[89] Negi NJ, Swanberg JE, Clouser JM, Harmon-Darrow C (2020). Working under conditions of social vulnerability: Depression among Latina/o immigrant horse workers. Cultur Divers Ethnic Minor Psychol.

[90] Humphrey J, Lindstrom M, Barton K, Shrestha P, Carlton E, Adgate J, Miller S, Root E (2019). Social and Environmental Neighborhood Typologies and Lung Function in a Low-Income, Urban Population. Int J Environ Res Public Health.

[91] Fujishiro K, MacDonald LA, Howard VJ (2020). Job complexity and hazardous working conditions: How do they explain educational gradient in mortality?. J Occup Health Psychol.

[92] Lin J, Rodriguez S, Zhou H, Sparks AD, Simmens SJ, Amdur R (2019). Racial disparities in late-stage prostate cancer: A SEER database analysis 2005–2015. JCO.

[93] Amini A, Jones B, Yeh N, Guntupalli S, Kavanagh B, Karam S, Fisher C (2016). Disparities in disease presentation in the four screenable cancers according to health insurance status. Public Health.

[94] Yang DX, Soulos PR, Davis B, Gross CP, Yu JB (2018). Impact of Widespread Cervical Cancer Screening: Number of Cancers Prevented and Changes in Race-specific Incidence. Am J Clin Oncol.

[95] Saumoy M, Schneider Y, Shen N, Kahaleh M, Sharaiha RZ, Shah SC (2018). Cost Effectiveness of Gastric Cancer Screening According to Race and Ethnicity. Gastroenterology.

[96] Roesch E, Amin A, Gupta J, García-Moreno Claudia (2020). Violence against women during covid-19 pandemic restrictions. BMJ.

[97] Allen-Ebrahimian B (2020). China's domestic violence epidemic. Axios.

[98] Taub A (2020). A new Covid-19 crisis: Domestic abuse rises worldwide. The New York Times.

[99] Peterman A, Potts A, O'Donnell M, Thompson K, Shah N, Oertelt-Prigione S, van Gelder N (2020). Pandemics and violence against women and children.

[100] WHO, Department of Reproductive Health and Research, London School of Hygiene and Tropical Medicine, South African Medical Research Council (2013). Global and regional estimates of violence against women: Prevalence and health effects of intimate partner violence and nonpartner sexual violence. World Health Organization.

[101] (2020). Work experience-people 15 years old and over, by total money earnings, age, race, Hispanic origin, sex, and disability status. United States Census Bureau.

[102] Levinson KL, Jernigan AM, Flocke SA, Tergas AI, Gunderson CC, Huh WK, Wilkinson-Ryan I, Lawson PJ, Fader AN, Belinson JL (2016). Intimate partner violence and barriers to cervical cancer screening: A gynecologic oncology fellow research network study. Journal of Lower Genital Tract Disease.

[103] Massetti GM, Townsend JS, Thomas CC, Basile KC, Richardson LC (2018). Healthcare Access and Cancer Screening Among Victims of Intimate Partner Violence. J Womens Health (Larchmt).

[104] Coker AL, Hopenhayn C, DeSimone CP, Bush HM, Crofford L (2009). Violence against Women Raises Risk of Cervical Cancer. J Womens Health (Larchmt).

[105] Alcalá Héctor E, Mitchell E, Keim-Malpass J (2017). Adverse Childhood Experiences and Cervical Cancer Screening. J Womens Health (Larchmt).

[106] Reingle Gonzalez JM, Jetelina KK, Olague S, Wondrack JG (2018). Violence against women increases cancer diagnoses: Results from a meta-analytic review. Prev Med.

[107] Guillaume E, Launay L, Dejardin O, Bouvier V, Guittet L, Déan Pauline, Notari A, De Mil R, Launoy G (2017). Could mobile mammography reduce social and geographic inequalities in breast cancer screening participation?. Prev Med.

[108] O'Donoghue D, Sheahan K, MacMathuna P, Stephens RB, Fenlon H, Morrin M, Mooney J, Fahy LE, Mooney T, Smith A (2019). A National Bowel Cancer Screening Programme using FIT: Achievements and Challenges. Cancer Prev Res.

[109] Levin TR, Corley DA, Jensen CD, Schottinger JE, Quinn VP, Zauber AG, Lee JK, Zhao WK, Udaltsova N, Ghai NR, Lee AT, Quesenberry CP, Fireman BH, Doubeni CA (2018). Effects of Organized Colorectal Cancer Screening on Cancer Incidence and Mortality in a Large Community-Based Population. Gastroenterology.

[110] Jessup DL, Glover Iv McKinley, Daye D, Banzi L, Jones P, Choy G, Shepard JO, Flores EJ (2018). Implementation of Digital Awareness Strategies to Engage Patients and Providers in a Lung Cancer Screening Program: Retrospective Study. J Med Internet Res.

[111] Harty NM, Le Grice K, Cahill C, Bull S, Dwyer A (2018). EndCancer: development and pilot testing of multimedia recruitment for a text message campaign to increase cancer screening. Mhealth.

[112] Guo T, Fan Y, Chen M, Wu X, Zhang L, He T, Wang H, Wan J, Wang X, Lu Z (2020). Cardiovascular Implications of Fatal Outcomes of Patients With Coronavirus Disease 2019 (COVID-19). JAMA Cardiol.

[113] Xia Y, Jin R, Zhao J, Li W, Shen H (2020). Risk of COVID-19 for patients with cancer. The Lancet Oncology.

[114] Zhang L, Zhu F, Xie L, Wang C, Wang J, Chen R, Jia P, Guan H, Peng L, Chen Y, Peng P, Zhang P, Chu Q, Shen Q, Wang Y, Xu S, Zhao J, Zhou M (2020). Clinical characteristics of COVID-19-infected cancer patients: a retrospective case study in three hospitals within Wuhan, China. Ann Oncol.

[115] Ahmed F, Ahmed N, Pissarides C, Stiglitz J (2020). Why inequality could spread COVID-19. The Lancet Public Health.

[116] Kuderer N, Choueiri T, Shah D, Shyr Y, Rubinstein Sm, Rivera Dr, Shete S, Hsu C, Desai A, de Lima Lopes G, Grivas P, Painter Ca, Peters S, Thompson Ma, Bakouny Z, Batist G, Bekaii-Saab T, Bilen Ma, Bouganim N, Larroya Mb, Castellano D, Del Prete Sa, Doroshow Db, Egan Pc, Elkrief A, Farmakiotis D, Flora D, Galsky Md, Glover Mj, Griffiths Ea, Gulati Ap, Gupta S, Hafez N, Halfdanarson Tr, Hawley Je, Hsu E, Kasi A, Khaki Ar, Lemmon Ca, Lewis C, Logan B, Masters T, McKay Rr, Mesa Ra, Morgans Ak, Mulcahy Mf, Panagiotou Oa, Peddi P, Pennell Na, Reynolds K, Rosen Lr, Rosovsky R, Salazar M, Schmidt A, Shah Sa, Shaya Ja, Steinharter J, Stockerl-Goldstein Ke, Subbiah S, Vinh Dc, Wehbe Fh, Weissmann Lb, Wu Jt, Wulff-Burchfield E, Xie Z, Yeh A, Yu Pp, Zhou Ay, Zubiri L, Mishra S, Lyman Gh, Rini Bi, Warner Jl, Abidi M, Acoba Jd, Agarwal N, Ahmad S, Ajmera A, Altman J, Angevine Ah, Azad N, Bar Mh, Bardia A, Barnholtz-Sloan J, Barrow B, Bashir B, Belenkaya R, Berg S, Bernicker Eh, Bestvina C, Bishnoi R, Boland G, Bonnen M, Bouchard G, Bowles Dw, Busser F, Cabal A, Caimi P, Carducci T, Casulo C, Chen Jl, Clement Jm, Chism D, Cook E, Curran C, Daher A, Dailey M, Dahiya S, Deeken J, Demetri Gd, DiLullo S, Duma N, Elias R, Faller B, Fecher La, Feldman Le, Friese Cr, Fu P, Fu J, Futreal A, Gainor J, Garcia J, Gill Dm, Gillaspie Ea, Giordano A, Glace (G, Grothey A, Gulati S, Gurley M, Halmos B, Herbst R, Hershman D, Hoskins K, Jain Rk, Jabbour S, Jha A, Johnson Db, Joshi M, Kelleher K, Kharofa J, Khan H, Knoble J, Koshkin Vs, Kulkarni Aa, Lammers Pe, Leighton Jc, Lewis Ma, Li X, Li A, Lo Ks, Loaiza-Bonilla A, LoRusso P, Low Ca, Lustberg Mb, Mahadevan D, Mansoor A, Marcum M, Markham Mj, Handy Marshall C, Mashru Sh, Matar S, McNair C, McWeeney S, Mehnert Jm, Menendez A, Menon H, Messmer M, Monahan R, Mushtaq S, Nagaraj G, Nagle S, Naidoo J, Nakayama Jm, Narayan V, Nelson Hh, Nemecek Er, Nguyen R, Nuzzo Pv, Oberstein Pe, Olszewski Aj, Owenby S, Pasquinelli Mm, Philip J, Prabhakaran S, Puc M, Ramirez A, Rathmann J, Revankar Sg, Rho Ys, Rhodes Td, Rice Rl, Riely Gj, Riess J, Rink C, Robilotti Ev, Rosenstein L, Routy B, Rovito Ma, Saif Mw, Sanyal A, Schapira L, Schwartz C, Serrano O, Shah M, Shah C, Shaw G, Shergill A, Shouse G, Soares Hp, Solorzano Cc, Srivastava Pk, Stauffer K, Stover Dg, Stratton J, Stratton C, Subbiah V, Tamimi R, Tannir Nm, Topaloglu U, Van Allen E, Van Loon S, Vega-Luna K, Venepalli N, Verma Ak, Vikas P, Wall S, Weinstein Pl, Weiss M, Wise-Draper T, Wood Wa, Xu W(, Yackzan S, Zacks R, Zhang T, Zimmer Aj, West J (2020). Clinical impact of COVID-19 on patients with cancer (CCC19): a cohort study. The Lancet.

[117] Sirintrapun SJ, Lopez AM (2018). Telemedicine in Cancer Care. American Society of Clinical Oncology Educational Book.

[118] Fertleman C, Aubugeau-Williams P, Sher C, Lim A, Lumley S, Delacroix S, Pan X (2018). A Discussion of Virtual Reality As a New Tool for Training Healthcare Professionals. Front Public Health.

[119] Mette L, Saldívar Anna Maria Pulido, Poullard N, Torres I, Seth S, Pollock B, Tomlinson G (2016). Reaching high-risk underserved individuals for cancer genetic counseling by video-teleconferencing. J Community Support Oncol.

[120] Strehle EM, Shabde N (2006). One hundred years of telemedicine: does this new technology have a place in paediatrics?. Arch Dis Child.

[121] (2010). Telemedicine: Opportunities and developments in member states. World Health Organization.

[122] Lewis GD, Hatch SS, Wiederhold LR, Swanson TA (2020). Long-Term Institutional Experience With Telemedicine Services for Radiation Oncology: A Potential Model for Long-Term Utilization. Adv Radiat Oncol.

[123] Ochs M, Mestre D, de Montcheuil G, Pergandi J, Saubesty J, Lombardo E, Francon D, Blache P (2019). Training doctors’ social skills to break bad news: evaluation of the impact of virtual environment displays on the sense of presence. J Multimodal User Interfaces.

[124] Lopez AM, Alberts DS, Hess LM (2019). Telemedicine, telehealth, and e-Health technologies in cancer prevention. Fundamentals of Cancer Prevention.

[125] Mendu S, Boukhechba M, Gordon J, Datta Debajyoti, Molina Edwin, Arroyo Gloria, Proctor Sara K, Wells Kristen J, Barnes Laura E (2018). Design of a Culturally-Informed Virtual Human for Educating Hispanic Women about Cervical Cancer. Proceedings of the 12th EAI International Conference on Pervasive Computing Technologies for Healthcare.

[126] Liang B, Yang N, He G, Huang P, Yang Y (2020). Identification of the Facial Features of Patients With Cancer: A Deep Learning-Based Pilot Study. J Med Internet Res.

[127] (2020). The rise of the data-driven physician. Stanford University.

[128] Lee A, Moy L, Kruck SE, Rabang J (2014). The doctor is in, but is academia? Re-tooling IT education for a new era in healthcare. Journal of Information Systems Education.

[129] Carroll N, Richardson I, Moloney M, O’Reilly P (2017). Bridging healthcare education and technology solution development through experiential innovation. Health Technol.

[130] Botrugno C (2019). Information technologies in healthcare: Enhancing or dehumanising doctor-patient interaction?. Health (London).

[131] Bidgoli H (2018). Successful Integration of Information Technology in Healthcare: Guides for Managers. JSIS.

[132] (2019). Mobile fact sheet. Pew Research Center.

[133] (2018). mHealth Economics 2017/2018 – Connectivity in Digital Health. Research 2 Guidance.

[134] Gordon WJ, Landman A, Zhang H, Bates DW (2020). Beyond validation: getting health apps into clinical practice. NPJ Digit Med.

[135] Noar SM, Harrington NG, Aldrich RS (2016). The Role of Message Tailoring in the Development of Persuasive Health Communication Messages. Annals of the International Communication Association.

[136] Wakefield MA, Loken B, Hornik RC (2010). Use of mass media campaigns to change health behaviour. The Lancet.

[137] Key TM, Czaplewski AJ (2017). Upstream social marketing strategy: An integrated marketing communications approach. Business Horizons.

[138] Patti C, Hartley S, van Dessel M, Baack D (2015). Improving integrated marketing communications practices: A comparison of objectives and results. Journal of Marketing Communications.

[139] Merrill RM, Telford CT (2018). Pharmaceutical use according to participation in worksite wellness screening and health campaigns. Prev Med Rep.

[140] Springer SM, McFall A, Hager P, Percy-Laury A, Vinson CA (2018). Lung cancer screening: an emerging cancer control issue presents opportunities for an awareness campaign in rural Michigan. Cancer Causes Control.

[141] Shaukat A, Church T (2020). Colorectal cancer screening in the USA in the wake of COVID-19.

[142] Nodora J, Gupta S, Howard N, Motadel Kelly, Propst Tobe, Rodriguez Javier, Schultz James, Velasquez Sharon, Castañeda Sheila F, Rabin Borsika, Martínez María Elena (2020). The COVID-19 Pandemic: Identifying Adaptive Solutions for Colorectal Cancer Screening in Underserved Communities. J Natl Cancer Inst.

[143] Del Vecchio Blanco G, Calabrese E, Biancone L, Monteleone G, Paoluzi OA (2020). The impact of COVID-19 pandemic in the colorectal cancer prevention. Int J Colorectal Dis.

